# Supplementary material for: A combined monthly precipitation prediction method based on CEEMD and improved LSTM
Source: PLoS One. 2023 Jul 13;18(7):e0288211. doi: 10.1371/journal.pone.0288211 (PMC10343162; doi:10.1371/journal.pone.0288211)
Supplement: Attachment — Submitted filename: Response to Reviewers.docx [file pone.0288211.s001.docx]

**Response to Reviewers**

**Title:** A combined monthly precipitation prediction method based on CEEMD and improved LSTM

**Thank you for your review comments. We appreciate your opinions and suggestions on our research. We have carefully read your review comments and made revisions and improvements to the article based on your suggestions.**

**We have conducted an in-depth analysis and discussion on the issues you raised, and have taken corresponding measures to revise and improve the article. We believe that after our efforts, the quality of the article has been significantly improved.**

**We greatly appreciate your review comments and assistance, which have been extremely valuable to our research. Thank you once again for your support and help!**

**Reviewer I**

1. Refine your innovation in Introduction Section.

***Response***

***We*** ***have fine-tuned*** ***the*** ***Introduction.The specific innovations are as follows:***

***For nonlinear and non-stationary data, the signal characteristics are usually complex and dynamic, making it difficult to effectively process and extract them using traditional linear analysis methods such as Fourier analysis and wavelet analysis. However, the CEEMD algorithm based on EMD can achieve adaptive decomposition of non-linear and non-stationary signals and decompose them into multiple IMFs, thereby better capturing the non-stationary and non-linear components contained in the signal.***

***In time series data, current data is often influenced by data from multiple previous time steps. Traditional feedforward neural networks cannot meet this requirement, resulting in errors in prediction. However, the design of long- and short-term memory units in LSTM networks allows for effective learning and memory of information from previous time steps, thereby better handling long-term dependencies and improving prediction performance.***

***Compared with other optimization algorithms, PSO (Particle Swarm Optimization) algorithm can quickly converge to the global optimal solution and usually has faster calculation speed than other algorithms. The PSO algorithm has good robustness and reliability, is less dependent on initial parameters, and is not easily trapped in local optimal solutions.***

2. Review of the literature can be improved further, may include the study of other applications of deep learning.

***Response***

***We have read the papers provided and appreciate your recommendation. We have cited and analyzed them in the introduction section.***

***[https://doi.org/10.1016/j.energy.2022.123990](https://doi.org/10.1016/j.energy.2022.123990)：Interpretable wind speed prediction with multivariate time series and temporal fusion transformers***

***The paper proposes a novel and interpretable wind speed prediction method, named VMD-ADE-TFT. This method may be innovative and unique in predicting wind speed and significantly improves the prediction accuracy. The introduced method in the paper may have good interpretability, which can help understand and explain the reasons and factors for the prediction results. The method presented in the paper can be applied to the field of wind speed prediction, which may have practical applications in the wind energy industry, weather forecasting and energy planning.***

3. Add MAPE to evaluate these model.

***Response***

***Thank you for your suggestion. We have added the MAPE index in the simulation part to analyze the predictive performance of the model.***

4.Why use PSO instead of other methods to optimize LSTM?

***Response***

***The particle swarm optimization (PSO) algorithm updates the position and velocity of each particle iteratively, gradually approaching the global optimal solution, thus having a fast convergence speed. Additionally, the parameter setting of PSO algorithm is relatively simple than some other optimization algorithms, which have mutual constraints among their parameters requiring fine tuning. Therefore, compared with other optimization algorithms, the PSO algorithm can converge to the global optimal solution quickly, and generally has a faster computation speed. The PSO algorithm has good robustness and reliability, with less dependence on initial parameters, making it less likely to fall into a local optimal solution.***

5.The Conclusions have not been written well. It is not concise and clear. It is better to explain briefly the research gaps, research objectives, and methodology, and then clearly present the main results obtained in the work.

***Response***

***Thank you for your suggestion. The conclusion section of the paper has been revised to briefly describe the research gaps, research objectives, and methodology, and present the main results obtained in the work.Specifically as follows:***

***Initially, the CEEMD decomposition algorithm was used to decompose the monthly precipitation with non-linearity and non-stationary characteristics into sub-sequences. Then, PSO was used to optimize the hyper-parameters of the LSTM network. Finally, the LSTM model was used to predict each sub-sequence, and the predicted results were combined to obtain the final monthly precipitation prediction.***

***The CEEMD decomposition method is used to decompose the monthly precipitation series, which reduces the interaction between different time scale information. Compared with EMD-LSTM and EEMD-LSTM methods, the RMSE of CEEMD-LSTM is reduced by 13.78 % and 3.75 % respectively, MAE is reduced by 6.21 % and 0.9 % respectively,and MAPE is reduced by 13.83% and 6.95% respectively. It shows that the CEEMD effectively improves prediction accuracy.***

***The PSO algorithm is used to optimize the hyper-parameters of the LSTM network, which avoids the contingency of manual selection. Compared with other optimization algorithms, PSO algorithm can quickly converge to the global optimal solution and have good robustness. LSTM network has certain superiority in handling time series data. Through the design of LSTM units, the LSTM network can effectively learn and remember information from previous time steps, thereby better handling long-term dependencies and improving prediction performance.***

***The combined CEEMD-PSO-LSTM model has been developed to effectively improve the accuracy of monthly precipitation prediction. The model is suitable for processing non-smooth, non-linear time-series data and can also be extended to the fields of electricity, traffic flow and text recognition.***

***In the next step, other influencing factors can be introduced, such as pressure, temperature, etc., to further improve the reliability and prediction accuracy of the model.***

**Reviewer** 2**:**

Question-I

Plz make Abstract informative and concise.

***Response***

 ***We*** ***have fine-tuned*** ***the Abstract.Specifically as follows:***

 ***With the continuous decline of water resources due to population growth and rapid economic development, precipitation prediction plays an important role in the rational allocation of global water resources. To address the non-linearity and non-stationarity of monthly precipitation, a combined prediction method based on complementary ensemble empirical mode decomposition (CEEMD) and a modified long short-term memory (LSTM) neural network was proposed. Firstly, the CEEMD method was used to decompose the monthly precipitation series into a set of relatively stationary sub-sequence components, which can better reflect the local characteristics of the sequence and further understand the nonlinear dynamic characteristics of the sequence. Then, improved LSTM neural networks were employed to predict each sub-sequence. The proposed improvement method optimized the hyper-parameters of LSTM neural networks using particle swarm optimization algorithm, which avoided the randomness of artificial parameter selection. Finally, the predicted results of each component were superimposed to obtain the final prediction result. The proposed method was validated by taking the monthly precipitation data from 1961 to 2020 in Changde City, Hunan Province as an example. The results of the case study show that, compared with other traditional prediction methods, the proposed method can better reflect the trend of precipitation changes and has higher prediction accuracy.***

Question-II

The advantages of CEEMD should be further clarified.

***Response***

 ***Thank you for your suggestion. We have supplemented the advantages of CEEMD in the introduction section.***

 ***The advantage of complementary ensemble empirical mode decomposition (CEEMD) is that it can effectively process non-linear and non-stationary signals and transform them into a group of relatively stable sub-sequence components, each representing signal components at different time scales. This decomposition method can better capture the inherent characteristics of the data and improve prediction accuracy. In addition, compared with other traditional time series analysis methods, CEEMD does not require prior assumptions about the specific form or basic functions of the signal, so it has strong adaptability and flexibility. Furthermore, CEEMD can automatically identify local features of the signal and effectively eliminates mode-mixing phenomena by decomposing a series of sub-sequences, making the amplitude and phase of each sub-sequence more closely resemble the original signal. This method can provide more accurate and reliable input data for prediction. Therefore, CEEMD, as an emerging time series analysis method, has a strong theoretical foundation and broad application prospects and is worth further exploration in future research and practice.***

Question-III

Section 3.2.3: Some studied of LSTM and applications should be added to highlight the advantages of LSTM ([https://doi.org/10.1016/j.energy.2022.126100](https://doi.org/10.1016/j.energy.2022.126100); Effective energy consumption forecasting using empirical wavelet transform and long short-term memory. Energy, 2022, 238: 121756; LSTM-Based Model Predictive Control of Piezoelectric Motion Stages for High-Speed Autofocus).

***Response***

 ***We have read the papers provided and appreciate your recommendation. We have cited and analyzed them in the introduction section.***

 ***Effective energy consumption forecasting using empirical wavelet transform and long short-term memory:***

 ***The model proposed in this paper achieves good prediction accuracy in energy consumption forecasting, exhibiting a relatively low mean absolute percentage error. LSTM is a type of recurrent neural network model that has advantages in processing time-series data and capturing long-term dependencies. The improved LSTM model applied in this study can better handle the sequential features of energy consumption data. The research emphasizes more critical influencing factors, which improves the accuracy and reliability of the prediction model.***

 ***LSTM-Based Model Predictive Control of Piezoelectric Motion Stages for High-Speed Autofocus:***

***Based on the LSTM model, this paper proposes an MPC method that successfully finds the optimal focus position through a series of focusing measurements from image sequences. Traditional internal controllers usually have difficulty in effectively dealing with the hysteresis effect of PEMA. By integrating the hysteresis effect into the model using LSTM units, this problem can be better addressed. By integrating the hysteresis effect and focusing measurements into a single learning model, this method can comprehensively consider various factors to improve the accuracy and performance of automatic focusing. To improve the speed of the LSTM-based MPC method, an optimized backpropagation algorithm is proposed in this paper to optimize the MPC cost function, further improving the efficiency of the algorithm. Moreover, this method is validated in experiments, and it can reduce automatic focusing time by at least 30% compared with the well-known rule-based automatic focusing methods and other learning-based methods.***

Question-IV

To improve the practicality, how to set the related parameters of LSTM for better performance?

***Response***

 ***LSTM is a commonly used type of recurrent neural network that consists of several key parameters such as input gates, forget gates and output gates. To obtain better performance, increasing the number of hidden neurons can improve the model's capacity, enhance its fitting ability, and help capture underlying patterns in the data. However, excessive numbers of neurons can result in over-fitting, so a suitable number needs to be chosen based on specific circumstances. Appropriate learning rate selection is also essential by considering factors like training data size and complexity. In this article, we mainly adopt the Particle Swarm Optimization algorithm to search for optimal hyper-parameters, and regularization techniques such as L1 and L2 regularization as well as dropout are used to improve the model's generalization ability and stability.***
